# Targeting Contrast Agents With Peak Near-Infrared-II (NIR-II) Fluorescence Emission for Non-invasive Real-Time Direct Visualization of Thrombosis

**DOI:** 10.3389/fmolb.2021.670251

**Published:** 2021-05-07

**Authors:** Kenneth S. Hettie

**Affiliations:** ^1^Department of Radiology, Stanford University School of Medicine, Stanford, CA, United States; ^2^Department of Otolaryngology - Head and Neck Surgery, Stanford University, Stanford, CA, United States

**Keywords:** thrombosis, occlusion, embolism, contrast agent, NIR-II fluorescence imaging

## Abstract

Thrombosis within the vasculature arises when pathological factors compromise normal hemostasis. On doing so, arterial thrombosis (AT) and venous thrombosis (VT) can lead to life-threatening cardio-cerebrovascular complications. Unfortunately, the therapeutic window following the onset of AT and VT is insufficient for effective treatment. As such, acute AT is the leading cause of heart attacks and constitutes ∼80% of stroke incidences, while acute VT can lead to fatal therapy complications. Early lesion detection, their accurate identification, and the subsequent appropriate treatment of thrombi can reduce the risk of thrombosis as well as its sequelae. As the success rate of therapy of fresh thrombi is higher than that of old thrombi, detection of the former and accurate identification of lesions as thrombi are of paramount importance. Magnetic resonance imaging, x-ray computed tomography (CT), and ultrasound (US) are the conventional non-invasive imaging modalities used for the detection and identification of AT and VT, but these modalities have the drawback of providing only image-delayed indirect visualization of only late stages of thrombi development. To overcome such limitations, near-infrared (NIR, ca. 700–1,700 nm) fluorescence (NIRF) imaging has been implemented due to its capability of providing non-invasive real-time direct visualization of biological structures and processes. Contrast agents designed for providing real-time direct or indirect visualization of thrombi using NIRF imaging primarily provide peak NIR-I fluorescence emission (ca. 700–1,000 nm), which affords limited tissue penetration depth and suboptimal spatiotemporal resolution. To facilitate the enhancement of the visualization of thrombosis *via* providing detection of smaller, fresh, and/or deep-seated thrombi in real time, the development of contrast agents with peak NIR-II fluorescence emission (ca. 1000–1,700 nm) has been recently underway. Currently, however, most contrast agents that provide peak NIR-II fluorescence emissions that are purportedly capable of providing direct visualization of thrombi or their resultant occlusions actually afford only the indirect visualization of such because they only provide for the (i) measuring of the surrounding vascular blood flow and/or (ii) simple tracing of the vasculature. These contrast agents do not target thrombi or occlusions. As such, this mini review summarizes the extremely limited number of targeting contrast agents with peak NIR-II fluorescence emission developed for non-invasive real-time direct visualization of thrombosis that have been recently reported.

## Introduction

Thrombosis (i.e., localized formation of a clot) in the vasculature can occur within the arterial or venous system when pathological factors compromise normal hemostasis, which is the complex process that maintains vascular integrity (e.g., in response to stasis, injury, or hypercoagulability) by regulating blood flow *via* platelet recruitment, fibrin formation, and blood coagulation (Rasche, 2001; Arnout et al., 2006; Tynngård et al., 2015; Stone et al., 2017; Yusof et al., 2019; Zhao Z. et al., 2020). Though both platelets and fibrin comprise both arterial and venous thrombi, the former tend to occur at sites of artery wall rupture (i) where wall shear rates are high in magnitude (ca. 100–100,000 s^–1^) and (ii) that are rich in platelets. Conversely, the latter tend to occur at sites of vein wall (i) where wall shear rates are low in magnitude (ca. 10–100 s^–1^) and (ii) that are rich in red blood cells (Jerjes-Sanchez, 2005; Lowe, 2008; Casa et al., 2015; Shi et al., 2016; Lippi and Favaloro, 2018; Chernysh et al., 2020; Delluc et al., 2020). Arterial thrombosis (AT) and venous thrombosis (VT) can lead to life-threatening cardio-cerebrovascular diseases (Hathcock James, 2006; Slevin and Krupinski, 2009; Chen et al., 2017). In particular, AT can lead to myocardial infarction (i.e., heart attack), thrombotic stroke, and ischemia, while VT can primarily lead to deep vein thrombosis, pulmonary embolism, femoral vein thrombosis, superior vena cava thrombosis, jugular vein thrombosis, cerebral venous sinus thrombosis, cavernous sinus thrombosis, portal vein thrombosis, and renal vein thrombosis (Lippi and Favaloro, 2018; Yusof et al., 2019). Importantly, the narrow therapeutic window immediately following the onset of AT and VT often does not allow for enough time to provide effective treatment (i.e., antiplatelet, anticoagulant, and/or thrombolytic therapy) (Smith et al., 2010; Yusof et al., 2019). As such, acute AT is the leading causes of heart attacks and approximately 80% of stroke incidences, both of which collectively constitute the most common cause of death, whereas the clinical ramifications of acute VT can be sudden death due to fatal complications from orally and systemically delivered therapeutics (Einhäupl et al., 1991; Wakefield, 2000; Attems et al., 2004; Abbott et al., 2007; Meissner et al., 2007; Mackman, 2008; Jackson, 2011; Previtali et al., 2011; Guirguis-Blake et al., 2016; Kim et al., 2016; Sidney et al., 2016; Stone et al., 2017; McCarthy et al., 2018). Accordingly, acute and chronic AT and VT are associated with high mortality and morbidity (typically due to a time lag between diagnosis and treatment, respectively), (Han et al., 2003; Smith et al., 2010; Hara et al., 2012; Go Alan et al., 2014; Singh et al., 2016; Stone et al., 2017). Accordingly, early diagnosis and treatment of thrombi could substantially reduce the risk of (i) thrombosis and the development of any resultant post-thrombotic complications (e.g., thrombi-induced occlusions) and (ii) hemorrhage/over-coagulation that can result from miscalculated therapy dosage (Scarvelis and Wells, 2006; Meissner et al., 2012). As the success rate of thrombolytic therapy in fresh thrombi (0–7 days in age) is significantly higher than that in old thrombi (7–10 days in age), accurate identification of fresh thrombi and early clinical treatment are crucial toward reducing both the rate of occurrence of and mortality from thrombus-induced cardio-cerebrovascular events (Ta et al., 2018).

In routine clinical practice, MRI, x-ray CT, and US are the conventional non-invasive imaging modalities used for the detection of arterial and venous thrombi (Moody, 2003; Grisham et al., 2005; Leach et al., 2006; Wiener et al., 2011; Hara et al., 2012; Gasparian et al., 2015; Gerstenhaber et al., 2017; Yusof et al., 2019). However, the capabilities of MRI, x-ray CT, and US are limited when utilized for this purpose (especially in emergency care) because these imaging modalities provide only image-delayed indirect visualization of (presumed to be) thrombi due to allowing for only (in this context) vasculature tracing and/or blood flow rate monitoring (Hara et al., 2012; Gerstenhaber et al., 2017). As MRI, x-ray CT, and US are inherently capable of indirectly reporting on lesion morphology only (as opposed to pinpoint lesion content), these imaging modalities are not capable of providing the (i) direct visualization of thrombi and (ii) necessary accurate identification of the pathological and physiopathological state of thrombi in real time (e.g., a bland versus a malignant thrombus) (Scarvelis and Wells, 2006; Hara et al., 2012; Gerstenhaber et al., 2017; Yusof et al., 2019). Importantly, as MRI, x-ray CT, and US also inherently suffer from affording relatively low sensitivity, high limits of detection, and/or poor spatiotemporal resolution, these imaging modalities are capable of detecting only late stages of thrombi development (i.e., old thrombi) and, thus, cannot detect fresh thrombi, which is crucial for addressing life-threatening acute AT and VT (Hara et al., 2012; Yusof et al., 2019). Similarly, though there are recent ongoing efforts in the development of tracers/probes for both the direct and indirect detection and identification of thrombosis for use in PET and single-photon emission computed tomography (SPECT), such ionizing radiation-based imaging modalities inherently provide worse resolution than MRI, x-ray CT, and US (Blasi et al., 2015; Kim et al., 2019; Hansen et al., 2020; Konstantinides, 2020; Le Roux et al., 2018). To overcome the collective shortcomings of all previously mentioned imaging modalities, near-infrared (NIR, ca. 700–1,700 nm) fluorescence (NIRF) imaging has recently emerged as a cost-effective technique for providing efficient non-invasive real-time direct visualization of biological structures and processes such that micro-/vascular lesions and vascular pathology content could be accurately diagnosed, respectively. NIRF imaging affords higher sensitivity, lower limits of detection, and higher spatiotemporal resolution (without the use of ionizing radiation) than those of MRI, x-ray CT, and US. Such attributes endow the optical imaging modality with the capability of promptly affording safe direct visualization of fresh thrombi as well as discrimination of their pathological and physiopathological condition (Gerstenhaber et al., 2017; Lim et al., 2017).

The overwhelming majority of contrast agents developed and/or utilized to facilitate either the direct or indirect visualization of thrombi *via* employing NIRF imaging provide peak fluorescence emission at wavelengths within the NIR-I spectral region (NIR-I, ca. 700–1,000 nm), whereby such contrast agents oftentimes consist of a Cy5.5, Cy7, or indocyanine green fluorophore to attain the deeper tissue penetration depths (ca. 1–10 mm) that NIR-I light affords as a result of it incurring both less photon absorption in tissue and scattering by biomolecules (e.g., hemoglobin) compared to fluorophores that provide peak fluorescence emission at wavelengths within the visible light spectrum (Vis, ca. 405–700 nm) (Frenette et al., 1995; Jaffer Farouc et al., 2002; Frangioni, 2003; Tung et al., 2003; Kim et al., 2005; Robert et al., 2007; Hara et al., 2012; Diao et al., 2015; Page et al., 2015; Vargas et al., 2015; Gerstenhaber et al., 2017; Lim et al., 2017; Kwon et al., 2018; Vankayala et al., 2018; Zhang et al., 2018; He et al., 2019; Lee et al., 2019; Yusof et al., 2019; Wan et al., 2020; Xu J. et al., 2020; Zhao Y. et al., 2020; Matsumura et al., 2021). The most recent advancement in the NIRF imaging of thrombosis and its sequelae is the development of contrast agents that provide peak fluorescence emission within the NIR-II optical window (NIR-II, ca. 1,000–1,700 nm), which can allow direct visualization of deep-seated thrombi because such emitted fluorescent light can achieve ∼3-fold deeper tissue penetration depths (ca. 10–30 mm) than contrast agents that provide peak NIR-I fluorescence emission (Lim et al., 2003; Wang W. et al., 2018; Ding F. et al., 2019; Hettie et al., 2019, 2020a,b; Sun Y. et al., 2019; Tu et al., 2019; Wang et al., 2019; Zhang et al., 2019; Li J. et al., 2020; Xu Y. et al., 2020). As a result, contrast agents that have peak fluorescence emission in the NIR-II spectral region afford lower limits of detection, higher sensitivity, and superior spatiotemporal resolution when imaging than contrast agents that emit fluorescent light with a peak wavelength within the visible and/or NIR-I spectral regions, and thereby provides the modality of NIRF imaging with such contrast agents the capability of enhanced and early detection of thrombi via affording unambiguous visualization and identification of previously hidden and/or fresh/smaller thrombi, respectively (Wan et al., 2020; Wu et al., 2020).

Currently, however, nearly all contrast agents that provide peak NIR-II fluorescence emission purportedly for use toward thrombosis imaging reported in the literature afford the indirect visualization of thrombi, non-occlusive thrombi, or occlusions due to such contrast agents only allowing for measuring of the blood flow rate or tracing of the vascular system similar to that provided by MRI, X-ray, and/or US; the thrombi or their resultant occlusions are not selectively or specifically targeted (Welsher et al., 2009; Semonin et al., 2010; Hong et al., 2012, 2014; Chen et al., 2014; Li et al., 2014, 2018, 2019; Antaris et al., 2016, 2017; Sun et al., 2016, 2017; Cosco et al., 2017; Kang et al., 2017; Lei et al., 2017, 2019; Yang et al., 2017, 2018, 2020; Zhong et al., 2017, 2019; Zhu et al., 2017; Ma et al., 2018; Shou et al., 2018; Wan et al., 2018; Wang P. et al., 2018; Zhang et al., 2018, 2020; Bai et al., 2019; Ding B. et al., 2019; Hu et al., 2019; Lin et al., 2019; Qu et al., 2019; Shi et al., 2019; Sun C. et al., 2019; Wang et al., 2019, 2020; Wu et al., 2019; Du et al., 2020; Li B. et al., 2020; Li D. et al., 2020; Li Y. et al., 2020; Liu et al., 2020; Tang et al., 2020; Tian et al., 2020; Zhou et al., 2020). Contrast agents that provide peak NIR-II fluorescence emission afford thrombi and occlusions to be indirectly visualized by (i) having previously acquired an image of the contrast agent-delineated vasculature prior to chemically treating or surgically inducing such manifestation and superimposing the pretreated or presurgical tracing over the resulting treated or postsurgical partial retracement, respectively, so as to discover the point of absence of a continuous contrast agent-demarcated vessel (i.e., a lesion), and/or (ii) simply having prior knowledge of the location of the chemically or surgically induced thrombus or occlusion and awaiting non-specific accumulation of the contrast agent (i.e., this route is effectively a byproduct of vascular tracing). In *arguendo*, for all intents and purposes, any such contrast agent that provides peak NIR-II fluorescence emission should likely produce similar results (i.e., vascular tracing) using such experimental technique(s) due to a contrast agent being non-specific. Accordingly, the goal of utilizing contrast agents with peak NIR-II fluorescence emission for NIRF imaging of thrombosis is to actually highlight thrombi/occlusions by targeting the lesions because visualization of vascular lesion could just as well be interpreted as an atherosclerotic plaque when said contrast agent is not accompanied by a targeting moiety. Accordingly, the contents of this mini review comprehensively summarize in detail the extremely limited number (i.e., 2) of targeting contrast agents with peak NIR-II fluorescence emission developed for non-invasive real-time direct visualization of thrombosis that serendipitously have been reported in the last few years.

## Luminescent Nanodots (NDs)

In mid-2020, Mateos et al. (2020) reported on a biocompatible colloidal suspension of silver sulfide (Ag_2_S) NDs (hydrophobic radius of ∼10 nm) that displays peak NIR-II fluorescence emission (ca. 1,200 nm) following functionalization with the octapeptide angiotensin II (Ang-II), which is the natural ligand for the angiotensin 1 receptor (AT1R), for selectively targeting ischemic myocardial tissue (i.e., coronary thrombosis) for its real-time direct visualization *in vivo via* using NIRF imaging. AT1R becomes highly expressed in the myocardium during a myocardial event. To achieve such an event, myocardial infarction was induced in a murine model by ligating its left descending coronary artery and performing reperfusion shortly thereafter. A murine sham surgery model (no infarct) was also prepared. The Ag_2_S NDs were administered systemically by their retro-orbital injection, and binding of the Ang-II functionalized NDs to the damaged area was monitored by capturing the NIR-II fluorescence emission every 5 min using an InGaAs camera (ca. 1,000–1,700 nm) upon irradiation by a 808 nm laser diode (power density = 0.2 W cm^–2^). To serve as a control, Ag_2_S NDs were functionalized with polyethylene glycol (PEG, *M*_w_ = 2,500 g mol^–1^) only that afforded identical Ag_2_S NDs to those of Ag_2_S-Ang II NDs (but lacking the Ang-II octapeptide), which similarly demonstrated a hydrophobic radius of ∼10 nm and peak NIR-II fluorescence emission at ∼1,200 nm.

**FIGURE 1:**
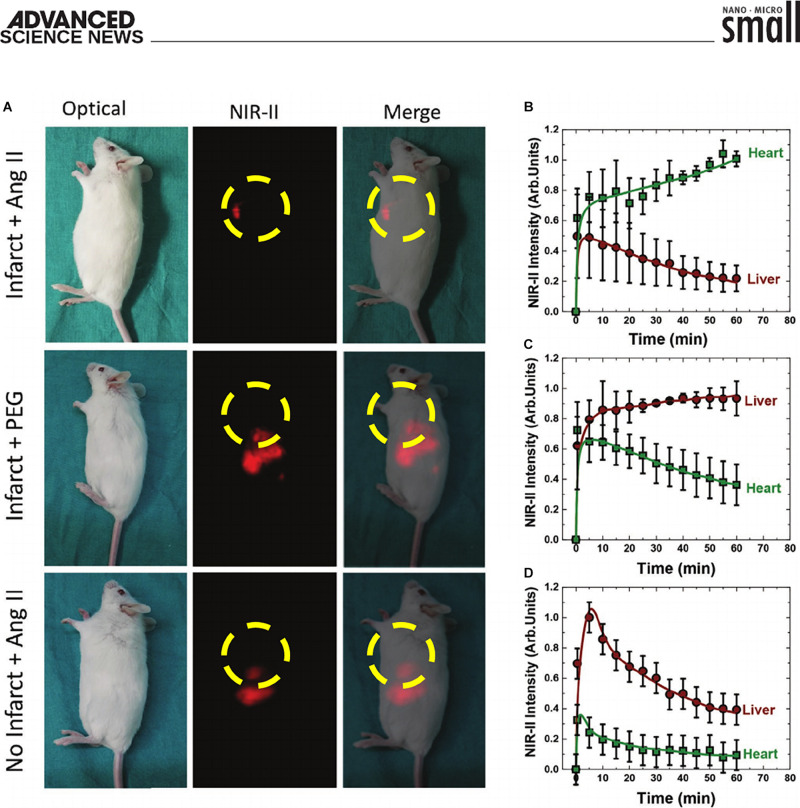
**(A)** Top row of panels: optical, NIR-II fluorescence, and merged image of targeting Ag_2_S-Ang II NDs (10 min post-administration) primarily accumulating at site of induced acute myocardial infarct in a murine model. Middle row of panels: optical, NIR-II fluorescence, and merged image of PEGylated-Ag_2_S NDs (10 min post-administration) non-specifically accumulating primarily at the liver. Bottom row of panels: optical, NIR-II fluorescence, and merged image of targeting Ag_2_S-Ang II NDs non-specifically accumulating primarily at the liver in a murine sham (i.e., no infarct) model. Imaged organ within yellow dashed circle highlights the heart. Please note that the location of the heart between images shifts due to the murine model being in a different rotated position. **(B–D)** Corresponding time course (60 min) of the NIR-II fluorescence intensity generated in the heart and liver following NDs administration. **(A–D)** Reproduced with permission (Mateos et al., 2020). Copyright 2020, WILEY-VCH Verlag GmbH & Co., KGaA, Weinheim.

The results revealed that Ag_2_S-Ang II NDs preferentially accumulated by adhering to the acutely damaged myocardial tissue, whereas, in absence of the infarct or when utilizing PEGylated-Ag_2_S NDs, no such accumulation at the infarcted heart occurred (Figure 1A, top panels). The Ag_2_S-Ang II NDs displayed nearly maximal accumulation within the first ∼10 min, which slowly trended upward over the course of the hour within which they were imaged (Figures 1B–D). To further bolster their findings that the NIR-II fluorescence signal from the images was indeed generated from the presence of the Ag_2_S-Ang II NDs, the hearts (infarcted and shams) were immediately resected following the 1 h time-course of the Ag_2_S NDs circulation (separately Ag_2_S-Ang II and PEGylated-Ag_2_S) and analyzed by hyperspectral imaging of NIR-II fluorescence using 1,000, 1,200, and 1,500 nm emission filter sets. Only the images of the infarcted heart from using only the 1,200 nm filter set when it was injected with the targeting Ag_2_S-Ang II NDs (as opposed to the non-targeting PEGylated-Ag_2_S NDs) afforded NIR-II fluorescence emission at the damaged myocardial tissue. The NIR-II fluorescence emission spectra from the hyperspectral cube effectively afforded a photophysical profile with no NIR-II fluorescence emission at 1,000 nm and 1,500 nm, but with the presence of peak NIR-II fluorescence emission at ∼1,200 nm. To demonstrate the overexpression of AT1R in the infarcted hearts, the Fan group performed a reverse transcriptase qualitative PCR (qtPCR) assay on both the infarcted and the sham hearts. The mRNA expression level of AT1R in the infarcted hearts displayed a ∼2-fold increase in AT1R expression compared to that of the sham hearts. An *ex vivo* biodistribution study was performed using the relevant organs, wherein only the heart and lungs from the infarct model using Ag_2_S-Ang II revealed any NIR-II fluorescence emission. However, only the liver and spleen from the infarct and sham models using PEGylated-Ag_2_S and Ag_2_S-Ang II, respectively, demonstrated NIR-II fluorescence emission.

The advantages of utilizing Ag_2_S NDs are their favorable biocompatibility, chemical stability and photostability, and high quantum yield. However, as the cores of Ag_2_S NDs are generally prepared by thermal decomposition and ligand exchange methods, they require extensive post-synthesis surface modification. For example, to afford favorable water-dispersion and long blood circulation half-life, the surface of Ag_2_S NDs needs to be engineered with a coating, such as PEG, of uniform consistency. Moreover, continuing with the previous example, subsequent functionalization of the PEG hydrophilic tail with a designated targeting moiety is required. Attaining homogeneity in size, coating, and the extent of targeting moiety functionalization are potential limitations of using Ag_2_S NDs, all of which could stifle their scale-up and successful clinical translation.

## Organic Small-Molecule Dyes (SMDs)

**FIGURE 2:**
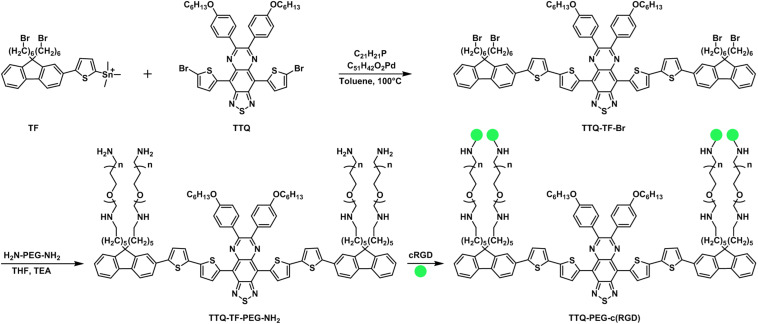
Synthetic scheme for TTQ-PEG-c(RGD).

In mid-2020, Wu et al. (2020) reported on an organic SMD composed of a 4,9-bis(5-bromothiophen-2-yl)-6,7-bis(4-(hexyloxy)phenyl)-[1,2,5] thiadiazolo[3,4-*g*]quinoxaline (TTQ)-based scaffold that was ultimately functionalized with cyclic tripeptide, cRGD, which targets activated platelet glycoprotein GPII/IIIa receptor (a receptor for fibrinogen), for use as a targeting contrast agent that provides peak NIR-II fluorescence emission for the selective direct visualization of thrombus *in vivo* (Figure 2). The SMD nanoparticle, referred to as TTQ-PEG-c(RGD), was prepared in three steps using TTQ as the starting material. The underlying framework of TTQ-PEG-c(RGD) was in the form of a donor–acceptor–donor (D–A–D) configuration, with PEGylated substituted fluorenes (that were appended onto TTQ by Stille couplings) serving as the electron donors and the centralized TTQ moiety serving as the electron acceptor, to afford it the capability of providing peak fluorescence emission in the NIR-II optical window. TTQ-PEG-c(RGD) displayed a wavelength of maximum absorbance at 750 nm and a wavelength of maximum fluorescence emission at 1,015 nm, whereby the quantum yield (ϕ_fl_) was measured to be ϕ_fl_ = 1.83% (IR1061 served as the reference) compared to single-walled carbon nanotubes (SWCNTs) and IR-20 measured to be ϕ_fl_ = 0.4% and ϕ_fl_ = 0.5%, respectively. The photostability of TTQ-PEG-c(RGD) demonstrated marginal attenuation in phosphate-buffered saline and fetal bovine serum when irradiated for an hour using an 808 nm laser. As the radius of the TTQ-PEG-c(RGD) was determined to be ∼40 nm, the hydrophobic radius was determined to be slightly larger ∼50 nm due to the four appended PEG moieties.

The cytotoxicity of TTQ-PEG-c(RGD) was determined. The SMD nanoparticle afforded a cell survival rate of ≥95% at varied concentrations, including a maximum mass concentration of 50 μg mL^–1^, thereby revealing that the SMD nanoparticle maintains a favorable safety profile. Murine thrombosis models *via* applying ferric chloride (FeCl_3_) to the external jugular vein were prepared. The murine thrombosis-induced models were imaged using a NIR-II imaging system outfitted with an InGaAs camera (1,000–1,700 nm) and 1,064 long-pass filter upon excitation at 808 nm using a diode laser (power density = 40 mW cm^–2^). Four hours after administration of TTQ-PEG-c(RGD) and a control nanoparticle, TTQ-TF-PEG-NH_2_, separately into murine thrombosis-induced models *in vivo*, a ∼7.6-fold relative difference in the NIR-II fluorescence emission intensities between the bilateral external jugular veins was attained when compared to the control group that received TTQ-TF-PEG-NH_2_. The specificity of TTQ-PEG-c(RGD) binding to the activated platelet glycoprotein GPII/IIIa receptor was verified by performing an inhibition study, wherein a cRGD inhibitor was administered prior to the administration of TTQ-PEG-c(RGD). An ∼4.8-fold relative difference in the NIR-II fluorescence emission intensities between the bilateral external jugular veins was attained when compared to the group that received the cRGD inhibitor. Importantly, TTQ-PEG-c(RGD) was able to discriminate between fresh thrombi and old thrombi. Murine thrombosis-induced models were prepared 5 days in advance, wherein such group of mice would serve as the old thrombus group. Freshly prepared murine thrombosis-induced models were also prepared, wherein this group of mice served as the fresh thrombus group. Four hours after administration of TTQ-PEG-c(RGD) into both the fresh and old thrombus group, an ∼3-fold relative difference in the NIR-II fluorescence emission intensity from the external jugular vein of the fresh thrombus group was achieved when compared to that of the old thrombus group. Collectively, TTQ-PEG-c(RGD) afforded the direct visualization of thrombosis in real time *in vivo* by targeting the activated platelet glycoprotein GPII/IIIa receptor with cRGD moieties. Moreover, TTQ-PEG-c(RGD) was capable of distinguishing fresh thrombus from old thrombus in murine thrombosis-induced models.

The advantages of utilizing SMDs as a scaffold for providing targeted NIR-II fluorescence imaging of thrombosis lies in the potential to easily engineer an endless array of flexible structures with tuned spectral and photophysical properties that are capable of readily undergoing targeting moiety modification. Currently, most SMDs utilize either the D–A–D or the polymethine structural format for achieving emission wavelengths in the NIR-II spectral region. The current drawback of utilizing these structural formats is the restriction of needing an underlying elongated conjugated core that has symmetry. As a result, the ability to modify the periphery of these structures or to attain an asymmetric core (which would be for the purpose of either developing such into a sensor or an OFF–ON probe) such that enhanced contrast levels could be achieved is not readily available.

## Discussion

The use of targeting contrast agents that afford peak NIR-II fluorescence emission to directly interrogate AT and VT in real time *via* NIRF imaging holds tremendous promise, due to providing higher sensitivity, lower limits of detection, and superior resolution, as well as direct visualization, when compared to the standard non-invasive clinical imaging modalities such as MRI, X-ray CT, and US. In addition, these targeting contrast agents that demonstrate peak NIR-II fluorescence emission for directly imaging thrombosis maintain characteristics that are more favorable than those that display peak fluorescence emission in the visible and NIR-I spectral regions that are designed for similar purposes. The use of contrast agents having peak fluorescence emission in the NIR-II optical imaging window can significantly aid in determining the identity of vascular lesions (e.g., a blood clot versus an atherosclerotic plaque) due to the inherent capabilities that NIRF imaging affords, namely the ability to target and discern vascular pathology content and processes as well as smaller thrombi. The recent advent of targeting contrast agents with peak NIR-II fluorescence emission to selectively and/or specifically identify biomarkers for direct visualization of thrombosis is a very new frontier that is ripe for significant advancements and acquisition of informative details in thrombosis, especially given the fact that an exhaustive review of the literature revealed that all of the contrast agents that provide peak NIR-II fluorescence emission purportedly for thrombosis imaging only allow for the indirect visualization of thrombi, non-occlusive thrombi, or occlusions, with the exception of two studies, which were set forth in detail in this comprehensive summary.

## Challenges, Conclusion, and Outlook

The challenges facing the ability to develop targeting contrast agents that have peak NIR-II fluorescence emission for thrombosis imaging include (i) limitations resulting from their spectral and photophysical profile(s) and (ii) the lack of diverse synthetic models that inhibit a means of extensive customization. Currently, though the fluorescence emission of NIR-II fluorescence contrast agents fall within the NIR-II spectral region, their wavelength of maximum absorbance oftentimes resides within the NIR-I spectral region, which limits their use in providing improved contrast levels and resolution due to the need for shorter excitation wavelengths of light. In addition, NIR-II fluorescence-emitting contrast agents typically display lower extinction coefficients as such contrast agents progressively emit longer wavelengths of light, which is a primary shortcoming of current SMDs. As low quantum yields typically also accompany such contrast agents, when coupled to their low extinction coefficients, the overall brightness of these contrast agents is poor. As such, an increased excitation power density can be utilized to help overcome these limitations, but such a workaround is feasible only to a limited extent before causing cellular damage. To overcome the limitations of current targeting contrast agents, new approaches toward their design are necessary. New structural formats and new design strategies that provide for versatile convenient engineering/synthesis would greatly expedite the realization of meeting unmet needs in thrombosis targeting and imaging. As for the clinical translation of NDs and SMDs, complex chemical engineering entails high costs and, as such, presents the need for simplicity in targeting contrasts agent development. Importantly, current clinical NIRF imaging instrumentation is outfitted with detectors/camera systems that provide for visualization of fluorescence emission in the Vis and NIR-I spectral regions. As such, both the development of targeting contrast agents for direct visualization of thrombosis using the NIR-II fluorescence imaging window and the NIR-I fluorescence imaging instrumentation itself are in the relatively early stages of development and use. However, as synthetic and technological advancements in these key areas are progressing at increasingly faster rates due to the extensive dissemination of scientific knowledge and an increase in scientific competition, the use of thrombosis-targeted contrast agents having NIR-II fluorescence emission in the clinic could soon become a reality.

## Author Contributions

KSH: conceptualization, background research, manuscript writing, editing, and submission.

## Conflict of Interest

The author declares that the research was conducted in the absence of any commercial or financial relationships that could be construed as a potential conflict of interest.
